# A systematic review on clinical guidelines of home health care in heart failure patients

**DOI:** 10.1186/s12912-023-01294-w

**Published:** 2023-04-18

**Authors:** Leila Hashemlu, Roghayeh Esmaeili, Fatemeh Bahramnezhad, Camelia Rohani

**Affiliations:** 1 PhD of Nursing, Student Research Committee, School of Nursing and Midwifery, Shahid Beheshti University of Medical Sciences, Tehran, Iran; 2 Department of Medical-Surgical Nursing, School of Nursing & Midwifery, Shahid Beheshti University of Medical Sciences, Tehran, Iran; 3 Nursing and Midwifery Care Research Center, School of Nursing and Midwifery, Tehran University of Medical Sciences, Tehran, Iran; 4 Spiritual Health Group, Research Center of Quran, Hadith and Medicine, Tehran University of Medical Sciences, Tehran, Iran; 5 Palliative Care Reseaech Center, Ersta Sköndal Bräcke University College, Campus Ersta, Stigbergsgatan 30, Box 11189, Stockholm, SE-100 61 Sweden; 6 Community Health Nursing Department, School of Nursing & Midwifery, Shahid Beheshti University of Medical Sciences, Tehran, Iran

**Keywords:** Clinical Guideline, Home Care, Heart failure patient, Systematic review

## Abstract

**Background:**

“Guidelines for the care of heart failure patients at home support safe and effective evidence-based practice. The aims of the present study were: [1] to identify guidelines addressing the care at home for adults with heart failure and [2] evaluate the quality of the guidelines and the extent to which they address eight components of home-based HF disease management.”

**Methods:**

A systematic review was conducted of articles published between 1st of January 2000 to 17th of May 2021 using the databases of PubMed, Web of Science, Scopus, Embase, Cochrane, and nine specific websites for guideline development organisations. Clinical guidelines for HF patients with recommendations relevant to care provision at home were included. The results were reported according to the Preferred Reporting Items for Systematic Reviews (PRISMA-2020) criteria. The quality of included guidelines was evaluated using the Appraisal of Guidelines for Research and Evaluation-II (AGREE-II) by two authors independently. Guidelines were evaluated for their coverage of eight components of HF care at home, consisting of integration, multi-disciplinary care, continuity of care, optimized treatment, patient education, patient and partner participation, care plans with clear goals of care, self-care management and palliative care.

**Results:**

Ten HF guidelines, including two nursing-focused guidelines and eight general guidelines were extracted from 280 studies. After evaluation of quality by AGREE-II, two guidelines obtained the highest score: “NICE” and the “Adapting HF guideline for nursing care in home health care settings. Five guidelines addressed all eight components of care at home while the others had six or seven.

**Conclusions:**

This systematic review identified ten guidelines addressing care at home for patients with HF. The highest quality guidelines most relevant to the care at home of patients with HF are the “NICE” and “Adapting HF guideline for nursing care in home health care settings” and would be most appropriate for use by home healthcare nurses.

**Supplementary Information:**

The online version contains supplementary material available at 10.1186/s12912-023-01294-w.

## Introduction

With growing numbers and complexity of persons living with HF, the management heart failure (HF) challenges the whole health system globally [1]. HF affects nearly 64.3 million people worldwide, a roughly two-fold increase from 33.5 million since 1990 [2]. The prevalence of HF has progressively increased for many years, both due to effective therapies keeping patients alive longer and the ageing of many populations worldwide [3], with the latter explaining more of the increase. Due to the chronic nature of HF, the recurrent disease exacerbations and patient’s re-admission to the hospital is one of the significant health problems in today’s society [4].

Multidisciplinary management of patients has been recommended in HF guidelines and it can improve caring outcomes [5]. A multidisciplinary team often consists of nurses, physicians, specialists in cardiology, in addition to physiotherapists, dieticians and social workers can provide standardized home care for HF patients [6, 7] by supporting person-centered care and self-management services. The person-centered approach to develop guidelines necessitates considering the patient’s conditions and needs, patient preferences, participation in goal-setting plans, and individual beliefs and values [8] and nurses are well positioned to support these functions.

Numerous studies show that ongoing person-centered care has positive results in nursing management of the HF patients and reduces their re-admission rate [9, 10]. One of the models for continuing care of the HF patients after discharging from hospital, is home healthcare services [11]. The term “home healthcare” can be perceived very different across countries according to their healthcare systems and delivering services for different target groups. Home healthcare services can deliver to the patients in their home or nursing home care centers. They can range from professional care for those requiring long-term care to those who only require assistance with relatively simple tasks on an as-needed basis (primarily support services or basic nursing care – e.g., bathing and dressing) [12]. Home healthcare services in patients’ home help families participate in their patient care and self-care. These services are a crucial component of community-based care services [10]. Maintaining and restoring patient’s independence is one of the primary missions of home healthcare services [13, 14].

Home healthcare nurses can provide services that previously were available only in hospitals. By transferring the knowledge and practice from hospitals to the patients’ home, the role of the home healthcare nurses is expanded [15]. HF patients receive different services at home by home healthcare nurses. Nurses manage patients in various aspects of physical, mental, spiritual and give them emotional support [16]. They provide general to specific care such as prevent medication and other medical errors, evaluate responses to therapy, identify early signs of problems such as impending volume overloading collaboration with patients’ physicians, implement strategies to prevent the onset of symptoms or minimize their effects, teach patients and caregivers about early symptom recognition, but also coach them about effective treatment, such as the use of as-needed diuretics at home for HF patients [7, 16, 17].

Nurses are one of the largest groups of the home-based healthcare providers, but provision of guideline-based caring has remains less than optimal [18]. Nursing management of HF patients at home is very critical. It has been recommended that the following eight components are considered: “integrated, multi-disciplinary care (integrate the care between community care, secondary and primary care, use a team approach, prioritize continuity of care and staff members), patient and partner participation, care plans with clear goals of care (focus care to improve quality of life, functional status and sense of security for patients include communication protocols palliative treatment), patient education (also family education), self-care management, appropriate access to care (use of tele-rehabilitation, telemonitoring, and telephone follow up, palliative care approach), optimize treatment (use guidelines and Individualize treatment” [19].

Clinical practice guidelines (CPGs) have helped to continuously improve patient safety and care across the globe. CPGs need to be both well developed and effectively introduced in clinical practice so that we can achieve quality patient care [20]. Although high-quality clinical guidelines can be a gold standard for practice [21], little is known about the content and consistency of HF guidelines relevant to homecare. To address these gaps, we conducted a systematic review with these aims: [1] to identify clinical home care guidelines in adult HF patients and their recommendations [2] to evaluate quality of the guidelines as well as to assess eight components of disease management at home in the guidelines.

**Table 1:** Characteristics of 10 selected clinical home care guidelines for HF patients in the study

Guideline (year)	Organisation	Country or region	Target users	Guideline writers	Standardized level of evidence	Search strategy for evidence
**Adapting heart failure guideline for nursing care in home health settings (2014)** **Update 2014** [27]	University of Pennsylvania, School of Nursing	USA	Home care nurses	Multidisciplinary	A, B, or C	Systematic literature review, modified the existing HFSA and AHA HF CPGs
**Practical guide on Home Health in HF patients (2012)** **Update 2012** [19]	University of Southern Denmark	Europe	Home care nurses, clinicians	Multidisciplinary	not mentioned	literature review, Survey of European heart failure management programmes, Opinion of researchers and practitioners
**Chronic heart failure in adults: diagnosis and management NICE** 1 **(2018)** **Update 2018** [28]	The National Collaborating Centre for Chronic Conditions/National Institute for Health and Clinical Excellence	United Kingdom	primary and secondary healthcare professionals	Multidisciplinary	Ia Ib IIa IIb III IV NICE 2	Systematic literature review
**Guidelines for the prevention, detection and management of chronic heart failure in Australia (2018)** **Update 2018** [29]	National Heart Foundation of Australia and Cardiac Society of Australia and New Zealand	Australia	general practitioners •general physicians, cardiologists, registrars and hospital resident medical officers •nurses and other allied health professionals •educators	Multidisciplinary	A, B, or C	Systematic literature review
**CCS** 3 **Canadian Cardiovascular Society Guidelines for the Management of Heart Failure (2017)** **Update 2021** [30]	Canadian Cardiovascular Society	Canada	clinicians, policymakers, and health systems	Multidisciplinary	High Quality EvidenceModerate Quality Evidence Low Quality Evidence	systematic review, primary and secondary panels
**Management of chronic heart failure SIGN** 4 **(2016)** **revalidated in 2019** [31]	Scottish Intercollegiate Guidelines Network	Scotland	healthcare professional cardiac nurses, cardiac surgeons, cardiologists, general practitioners, pharmacists, psychologists, patients, carers, voluntary organisations and policy makers	Multidisciplinary	1++ 1+ 1 – 2++ 2+ 2 – 3 4 5	Systematic literature review
**ESC** 6 **Guidelines for the diagnosis and treatment of acute and chronic heart failure (2016)** **Update 2016** [32]	European Society of Cardiology	Europe	Health professionals	Multidisciplinary	Classes of recommendations I II IIa IIb III LOE A, B, or C	Systematic literature review
**AHA /ACCF** 7 **Guideline for the Management of Heart Failure(2013)** **Update 2021** [33]	American College of Cardiology Foundation /American Heart Association (ACC/AHA)	USA^8^	physicians and nurse	Multidisciplinary	LOE^9^ A, B, or C^10^	Systematic literature review
**HFSA** 11 **Comprehensive Heart Failure Practice Guideline (2010)** **Update 2017 ACC/AHA/HFSA** [34]	Heart Failure Society of America	USA	physicians and a nurse	Multidisciplinary	LOE A, B, or C	Systematic literature review
**ICSI** 12 **Palliative Care for Adults (2020)** **Update 2020** [35]	Institute for Clinical Systems Improvement	USA	primary and specialty care providers	Multidisciplinary	High Quality Evidence Moderate Quality Evidence Low Quality Evidence	Systematic literature review

## Methods

The present systematic review is reported using the Preferred Reporting Items for Systematic Reviews (PRISMA 2020) [22]. The protocol of this systematic review was registered on PROSPERO (CRD42021241979).

### Data sources and search strategy

A systematic search was performed to identify appropriate guidelines published between the1st of January 2000 to 17th of May 2021. We did an extensive search in databases of PubMed, Web of Science, Scopus, Embase, Cochrane and nine specific websites for organizations of guideline development, including “Agency for Healthcare Research and Quality & National Guideline Clearinghouse, Guideline International Network (G-I-N), New Zealand Guidelines Group, National Health and Medical Research Council (NHMRC), National Institute for Clinical Excellence (NICE; UK), Australian National Health and Medical Research Council, Scottish Intercollegiate Guidelines Network (SIGN), Canadian Medical Association InfoBase of Clinical Practice Guidelines, Professional CR society websites (ICCPR; http://globalcardiacrehab.com/cr-guidelines/). In addition, authors carried out manual searches as a supplemental approach to identify additional primary studies for systematic reviews [23]. Appropriate keywords were identified using Medical Subject Headings (Mesh). The selected keywords were: guideline/guidelines or recommendation, or guideline adherence or practice guideline, and home care and heart failure. These keywords were combined together by Boolean operators, and an extensive search was done (Appendix1).

### Study selection

Two authors independently screened all potentially relevant studies by reading the titles, abstracts and full-text of the studies according to inclusion criteria of the study. Disagreements were solved by discussion and using the viewpoint of a third reviewer.

### Inclusion and exclusion criteria

The term “home care” can be perceived differently across countries, depending on their healthcare systems and how they deliver services to different target groups. The definition of home care used in this review includes short-term and long-term professional care provided by home healthcare nurses within own patients’ home [12]. The scope of home healthcare services for HF patients can be preventive, acute, rehabilitative or palliative.

The inclusion criteria for this study were: the guideline was developed specifically for patients with HF, and the publication language was English. It was published between 1st of January 2000 to 17th of May 2021, and was labelled guideline/guidelines, or recommendation, or guideline adherence or practice guideline. When there were multiple versions of the guideline, the most recently updated one was chosen.

The exclusion criteria were: the guidelines did not reference home health care services, it was not supported by a health professional association or society, public or private organization, healthcare organization or government agency. Also, it did not target HF patients, and its recommendations was not based on a systematic literature search.

**Table 2:** The results of quality evaluation of the clinical home care guidelines by using the AGREE-II

Domains	Adapting HF guideline for nursing care in home (2014) [27]	Practical guide on Home Health in HF patients (2012) [19]	NICE (2018) [28]	prevention, detection and management of HF Australia (2018) [29]	CCS (2017) Guidelines for the Management of HF (2017) [30]	SIGN (2016) [31]	ESC (2016) [32]	AHA / ACCF (2013) [33]	HFSA (2010) [34]	ICSI Palliative Care for Adults (2020) [35]	
**Domain 1: scope and purpose**	60	95	65	90	40	60	50	60	56	70	
**Domain 2: stakeholder involvement**	62	95	70	78	25	60	50	45	30	50	
**Domain 3: rigor of development**	71	25	84	60	76	79	73	78	50	85	
**Domain 4: clarity of presentation**	66	48	100	53	70	88	94	85	63	79	
**Domain 5: applicability**	58	30	55	39	50	40	39	42	40	39	
**Domain 6: editorial independence**	50	50	90	75	100	93	83	90	92	95	

### Data collection

**Fig. 1:**
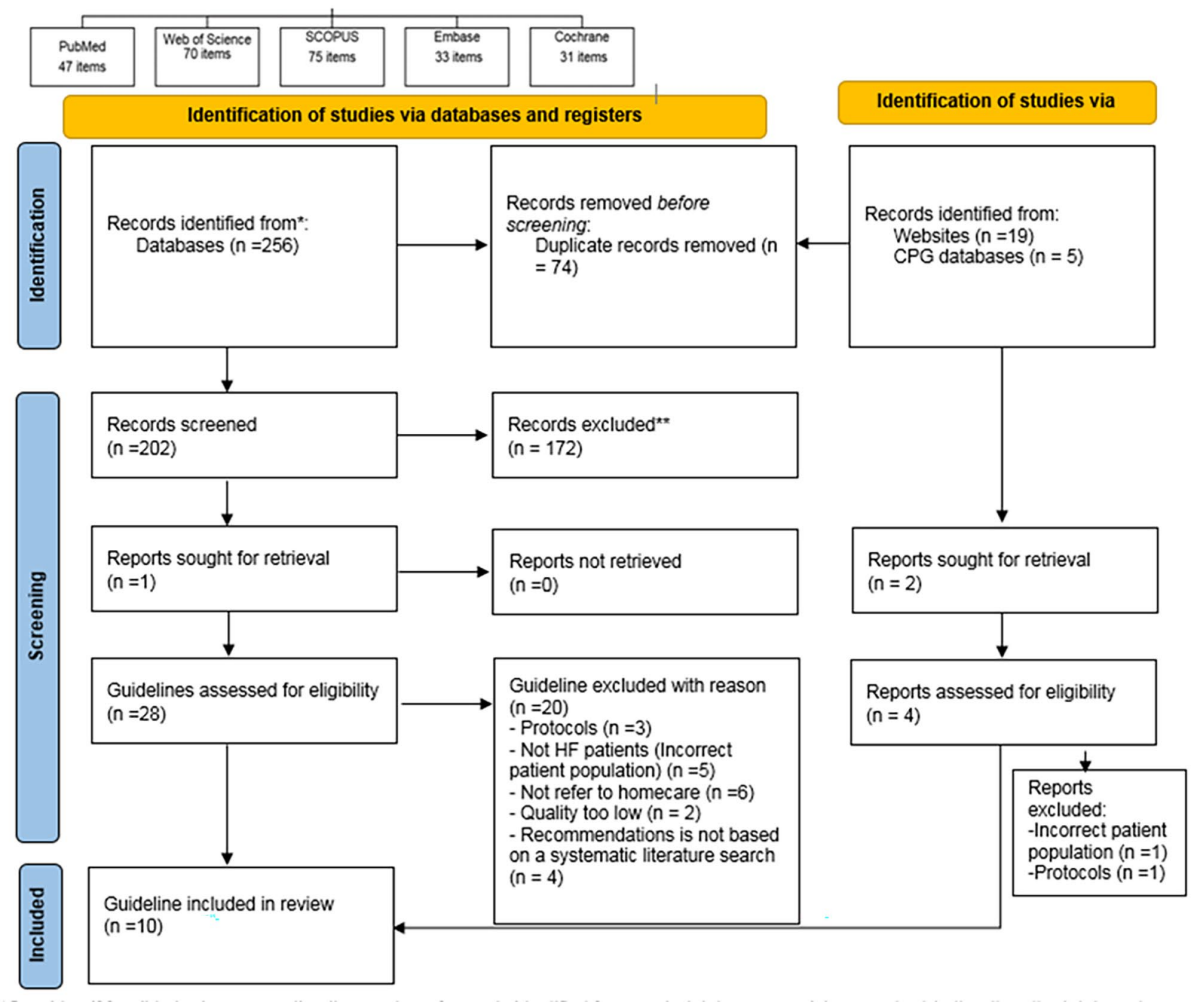
Diagram of the Study selection according to the PRISMA 2020 [25]

Data collection was divided into the three steps in our study: [1] to run a systematic search and selection of current evidence-based guidelines for HF patients that can be applied to home-based care (Fig. 1), [2] to evaluate of the methodological quality of the selected guidelines with the Appraisal of Guidelines for Research and Evaluation (AGREE-II) and [3] to compare recommendations of the guidelines with the eight components of disease management at home [19].

Two independent evaluators conducted data extraction. They used specific forms that were designed in the research team to extract the required data. When there was no consensus, a third evaluator assessed the situation. The data were extracted from all included guidelines: title, publication year, organization, country or region, target users, standardized level of evidence, search strategy for evidence. During the whole the process, a third evaluator checked the data for errors and resolved discrepancies or disagreements through discussions or consultations.

**Table 3:** Reported recommendations field in 10 selected clinical home care guidelines in HF patients

Title of guideline (year);	Recommendations Field; (number of items)
**Adapting heart failure guideline for nursing care in home health settings (2014)** [27]	• Integrated, multi-disciplinary care (6 items) • Patient and partner participation (4 items) • Care plans with clear goals of care (6 items) • Patient education(4 items) • Self-care management (3 items) • Appropriate access to care (3 items) • Optimize treatment (4 items)
**Practical guide on Home Health in HF patients (2012)** [19]	• HF Generic(Initial evalution, Serial clinical assessment of patient with HF, Nonpharmacologic Management and Health Care Maintenance in Patients With Chronic HF, Advance Directives and End-of-Life Care in HF) (50 items) • HF w normal EF(12 items) • HF with Red EF(63 items) • HF co-morbidities(4 items) • HF in minority pop(13 items)
**Chronic heart failure in adults: diagnosis and management NICE (2018)** [28]	• Team working in the management of HF(9 items) • Diagnosing HF(17 items) • Giving information to people with HF(5 items) • Treating HF with reduced ejection fraction(28 items) • Treating HF with reduced ejection fraction in people with chronic kidney disease(3 items) • Managing all types of HF(15 items) - Pharmacological treatment - Depression - Lifestyle(Salt and fluid restriction, Smoking, Alcohol, Sexual activity, Air travel, Driving regulations) • Monitoring treatment for all types of HF(5 items) • Interventional procedures(4 items) • Cardiac rehabilitation(1 item) • Palliative care(5 items)
**Guidelines for the prevention, detection and management of chronic heart failure in Australia (2018)** [29]	• Prevention of HF(3 items) • Diagnosis and Investigations(4 items) • Acute HF(9 items) • Pharmacological Management of Chronic HF - HF With Reduced Left Ventricular Ejection Fraction(3 items) - Selected Patients with HF with Reduced Left Ventricular Ejection Fraction(7 items) • Non-pharmacological management (Systems of Care to Reduce Rehospitalisation, Models of Care to Improve Evidence-Based Practice ,Multidisciplinary HF Disease Management Programs and Telemonitoring, Nurse-led Medication Titration Clinics, Non-pharmacological HF Management and Multimorbidity, Frequency of Follow-up, Self-management, Fluid Restriction and Daily Weighing, Sodium, Exercise Training and HF) (7 items) • Devices, Surgery and Percutaneous Procedures(6 items) • Comorbidities in HF(14 items) • Chemotherapy-related Cardiotoxicity and HF(5 items) • Treatment of HF With Recovered Ejection Fraction(1 item) • Special Situations(Driving, Travel, Vaccination, Sex, Pregnancy, Contraception, Caffeine Intake) (8 items) • Palliative Care in HF(1 item)
**CCS** **Canadian Cardiovascular Society Guidelines for the Management of Heart Failure (2017)** [30]	• Prevention of HF and Asymptomatic LV Dysfunction - Early detection of LVSD and prevention of HF(6 items) - Preventing HF in patients with hypertension(2 items) - Preventing HF in patients with diabetes(6 items) • Diagnosis of HF(6 items) • Biomarkers/NPs(5 items) • Treatment - Chronic HF - HFrEF pharmacological treatment(20 items) - HFpEF pharmacological treatment (4 items) - Implantable cardiac devices(14 items) - Advanced HF management strategies - mechanical circulatory support(7 items) - Exercise and rehabilitation (2 items) - Important nonpharmacological and nondevice management options(3 items) - Cardiovascular comorbidities(38 items) - Noncardiovascular comorbidities(16 items) - Acute HF(11 items) - Special circumstances(27 items) • Community Management of HF(8 items) • Quality Assurance/Improvement(2 items)
**Management of chronic heart failure SIGN (2016)** [31]	• Diagnostic investigations(2 items) • Emotional wellbeing and health behavior change(7 items) • Pharmacological therapies(20items) • Interventional procedure(5 items) • Post discharge care( Nurse-led follow, Role of pharmacists, Self-management) (3 items) • Palliative care( Prognosis and identifying patients with palliative care needs, Quality of life, Symptom management, Rationalizing treatments) (4 items) • Provision of information(Communication, Checklist for provision of information, Sources of further information) (3 items) • Implementing the guideline(4 items)
**ESC Guidelines for the diagnosis and treatment of acute and chronic heart failure (2016)** [32]	• Diagnosis(3 items) • Cardiac imaging and other diagnostic tests(10 items) • Delaying or preventing the development of overt HF or preventing death before the onset of symptoms(1 item) • Pharmacological treatment of HF with reduced ejection fraction(6 items) • Non-surgical device treatment of HF with reduced ejection fraction(3 items) • Treatment of HF with preserved ejection fraction(3 items) • Arrhythmias and conductance disturbances • Co-morbidities(17 items) • Acute HF(7 items) • Mechanical circulatory support and heart transplantation(2 items) • Multidisciplinary team management(Organization of care, Discharge planning, Lifestyle advice, Exercise training, Follow-up and monitoring, The older adult, frailty and cognitive impairment and end-of-life care) (7 items)
**AHA /** **ACCFGuideline for the Management of Heart Failure (2013)** [33]	• Initial and Serial Evaluation of the HF Patient (5 items) • Treatment of Stages A to D (4 items) - Stage A(Recognition and Treatment of Elevated Blood Pressure, Treatment of Dyslipidemia and Vascular Risk, Obesity and Diabetes Mellitus, Recognition and Control of Other Conditions That May Lead to HF) - Stage B(Management Strategies for Stage B) - Stage C(Nonpharmacological Interventions(Education, Social Support, Sodium Restriction, Treatment of Sleep Disorders, Weight Loss, Activity, Exercise Prescription, and Cardiac Rehabilitation), Pharmacological Treatment for Stage C) - Stage D(Definition of Advanced HF, Important Considerations in Determining If the Patient Is Refractory, Water Restriction, Inotropic Support, Mechanical Circulatory Support, Cardiac Transplantation) • The Hospitalized Patient(Inpatient and Transitions of Care) (9 items) • Important Comorbidities in HF(Atrial Fibrillation, Anemia,Depression,Other Multiple Comorbidities) (4 items) • Surgical/Percutaneous/Transcatheter Interventional Treatments of HF(1 item) • Coordinating Care for Patients With Chronic HF(Systems of Care to Promote Care, Coordination for Patients With Chronic HF, Palliative Care for Patients With HF) (3 items)
**HFSA Comprehensive Heart Failure Practice Guideline (2010)** [34]	• Development and Implementation of a Comprehensive HF Practice Guideline • Conceptualization and Working Definition of HF • Prevention of Ventricular Remodeling, Cardiac Dysfunction, and HF(4 items) • Evaluation of Patients for Ventricular Dysfunction and HF(21 items) • Management of Asymptomatic Patients With Reduced Left Ventricular Ejection Fraction(7 items) • Nonpharmacologic Management and Health Care Maintenance in Patients With Chronic HF(19 items) • HF in Patients With Reduced Ejection FractionDisease Management, Advance Directives, and End-of-Life Care in HF(41 items) • Electrophysiology Testing and the Use of Devices in HF(17 items) • Surgical Approaches to the Treatment of HFEvaluation and Management of Patients With HF and Preserved Left Ventricular Ejection Fraction(12 items) • Evaluation and Management of Patients With Acute Decompensated HF(7 items) • Evaluation and Therapy for HF in the Setting of Ischemic Heart Disease(10 items) • Managing Patients With Hypertension and HF(26 items) • Management of HF in Special Populations(16 items) • Managing Patients with Hypertension and HF (6 items) • Management of HF in Special Populations (11 items) • Myocarditis: Current Treatment (2 items) • Genetic Evaluation of Cardiomyopathy(8 items)
**ICSI Palliative Care for Adults (2020)** [35]	• Initiate Palliative Care Discussion (2 items) • Assess Patient’s Palliative Care Needs Based on the Following Domains of Palliative Care(4 items) • Begin Advance Care Planning Process(2 items) • Physical Aspects of Care • Cultural Aspects of Care • Psychological and Psychiatric Aspects of Care • Social Aspects of Care • Spiritual Aspects of Care • Ethical and Legal Aspects of Care (1 items) • Develop or Revise Palliative Care Plan and Establish Goals of Care through the Process of Shared Decision-Making (1 items) • Does Patient Meet Hospice Criteria? (1 items) • Care for the Dying Patient (1 items) • Grief and Bereavement

To evaluate the methodological quality of the guidelines, AGREE-II was used. The AGREE-II includes 23 items divided into the following six categories: scope and purpose (3 items), stakeholder involvement (3 items), the rigor of development (8 items), clarity of presentation (3 items), applicability (4 items) and editorial independence (2 items). A seven-point Likert scale is used to evaluate each one of the 23 items between 1 (strongly disagree) to 7 (strongly agree). Each of the six-domain scores is calculated separately by adding up all the scores of the specific items in a domain, as well as by calculating the aggregate as a percentage of the highest score for that domain. The following method was used to calculate the Domain scores (obtained score - minimum possible score) / (maximum possible score - minimum possible score). The minimum possible score was calculated as 1× (number of items) × (number of appraisers). The maximum possible score was calculated as 7× (number of items) × (number of appraisers) [24]. The value of 50% has been defined as a cut-off for AGREE-II, and values over that threshold were deemed satisfactory [25]. The quality assessment of all included clinical guidelines was performed by two evaluators, independently.

### Comparison of the clinical guidelines based on the eight components of HF management at home

In integrating hospital care to home, it has been advised to consider the following components in home care of HF patients: ”Integrated, multi-disciplinary care, continuity of care and care plans, optimized treatment according to guidelines, patients and caregiver’s education, patient and partner participation, care plans with clear goals of care, self-care management, and palliative care” [19, 26]. We used these components to identify comprehensive guidelines for home care of HF patients.

## Results

The results of study selection were shown based on the PRISMA 2020 [26] in Fig. 1. In the initial search, 280 records were obtained. From 206 non-duplicate records, the title and abstract of each study were screened, of which 174 were excluded and, 32 full guidelines text remained; among, them, 22 guidelines were excluded due to incorrect patient population, protocols, not refer to homecare, quality too low, not based on a systematic literature search were excluded and, the final selection yielded a total of 10 clinical practice guidelines for HF patients, including two nursing-focused guidelines [19, 27] and eight general guidelines [28–35].

### Characteristics of the clinical guidelines

Table 1. presents the characteristics of the guidelines included. The majority (60%) of the guidelines were published or updated within the latest three years. Among the 10 guidelines, seven (70%) were developed or published by national institutions of HF, and the remaining three by the independent expert panel and Institute for Clinical Systems Improvement (ICSI). Overall, all of guidelines were developed based on evidence (100%). The guidelines were developed in different places: the USA (four guidelines), the UK (one guideline), Europe (two guidelines), Canada (one guideline), Scotland (one guideline), and Australia (one guideline).

### Quality assessment of guidelines

Quality assessment of guidelines was done based on the AGREE-II guidelines. The AGREE-II includes 23 items divided into the following six domains: scope and purpose (3 items), stakeholder involvement (3 items), the rigor of development (8 items), clarity of presentation (3 items), applicability (4 items) and editorial independence (2 items).The results of the domain scores of the 10 guidelines are shown in Table 2. Among the 10 guidelines, guidelines of “the National Institute for Health and Care Excellence -NICE” and “Adapting HF Guideline for Nursing Care in Home Healthcare settings scored higher than 50% across all six domains. The field of Recommendations for all 10 selected guidelines are shown in Table 3.

### Evaluation of eight components of disease management at home in the guidelines

Eight principal components of HF patient’s management at home were evaluated in all guidelines. They were extracted from a practical home care guide for HF patients in the guidelines following a systematic review and an international expert panel meeting [19, 26]. Our results showed that the level of details varied in the guidelines. Five guidelines addressed all eight components and the rest of them addressed six or seven components. These results are shown in Table 4.

**Table 4:** Comparison of the clinical guidelines based on the recommended components of home care

Components of home care	Adapting HF guideline for nursing care in home (2014) [27]	Practical guide on Home Health in HF patients (2012) [19]	NICE (2018) [28]	prevention, detection and management of HF Australia (2018) [29]	CCS (2017) Guidelines for the Management of HF (2017) [30]	SIGN (2016) [31]	ESC (2016) [32]	AHA / ACCF (2013) [33]	HFSA (2010) [34]	ICSI Palliative Care for Adults (2020) [35]	
**Integrated, multi-disciplinary care**	•	•	•	•	•	•	•	•	•	•	
**continuity of care and care plans**		•		•	•			•		•	
**optimized treatment according to guidelines**	•	•	•	•	•	•	•	•	•	•	
**patients and caregivers education**	•	•	•	•	•	•	•	•	•	•	
**Patient and partner participation**	•	•	•	•	•	•	•	•	•	•	
**Care plans with clear goals of care**		•	•	•	•	•	•	•	•	•	
**Self-care management**	•	•	•	•	•	•	•	•	•	•	
**Appropriate access to care (palliative care, tele-monitoring)**	•	•	•	•	•	•	•	•	•	•	

## Discussion

This is the first systematic review to identify the quality of clinical practice guidelines on home-based care for HF Patients. In this review, two nursing-focused guidelines and eight general guidelines were extracted. All general HF guidelines can be applied to HF care at home, depending on the clinical characteristics and the need for interprofessional HF training as well as more attention to home care planning and advanced care. Our results showed that there are two specific HF CPGs for home care nursing.

The first specific HF guideline was “Practical guide on Home Health in HF patients” (2012) [19]. The purpose of this guide was to describe the characteristics of home-based heart failure care and develop guidance for establishing and delivering home-based care for HF patients by health care providers. One of the preferences of this guide was including eight components of HF care at home; Integrated, multi-disciplinary care, continuity of care and care plans, optimized treatment according to guidelines, patients and caregiver’s education, patient and partner participation, care plans with clear goals of care, self-care management, and palliative care. In our study, we used these components to categorize all selected guidelines. The second CPGs was “adapting HF guideline for nursing care in home health settings” that adapt general HF CPGs for home health nursing expectations and scope of practice [27].

CPGs’ quality, detail of recommendations, and applicability vary, making selecting high-quality CPGs to implement complex. Based on the results of the study, nurses should be aware of the differences in the quality between these guidelines and try to use the highest quality guidelines based on the context and health system. The first step in improving the quality and outcomes for HF patients receiving home care is to identify clinical home care guidelines for adult HF patients and their recommendations, evaluate the quality of the guidelines, and assess eight components of disease management at home in the guidelines. CPGs should create various materials to support implementation activity and offer advice on implementing the recommendations [36]. Therefore, we recommend that nurses rely on CPGs that perform better in the ‘applicability’ domain.

By using the AGREE-II, the quality of all included guidelines were evaluated. AGREE-II assesses how well a CPG development process is reported, but the content of the CPG recommendations has not been reported. We have attempted to consider capturing this information detail within our extraction of guideline recommendations and Comparison of the clinical guidelines based on the recommended eight components of home care [19]. This study was similar to previous systematic evaluations of clinical practice guidelines in other clinical disciplines: the highest average AGREE-II values were computed in domains of “editorial independence” and “clarity of presentation” In contrast, the lowest average score was acquired in the domain of “Applicability” [37]. A large majority of guidelines were developed without considering if they had recognized facilitators and obstacles to execution, presented criteria for monitoring or auditing, conducted economic analysis, and provided practice instruments.

HF CPGs were rated based on the AGREE-II in our study. “Chronic heart failure in adults: diagnosis and management NICE-2018” and “adapting HF guideline for nursing care in home health settings guidelines” achieved score of more than 50% in all six domains. NICE guidelines used evidence-based strategies that weighed possible opportunities and risks, as well as clinical and cost-effectiveness. Besides, during the guideline development process, NICE involved multi-disciplinary guideline workgroups, including stakeholders in a collaborative, explicit, and transparent manner. It produced a range of materials to support implementation activity [28].

All guidelines can be a valuable guide for health care professionals who are involved in the home care of HF patients, thereby reducing unnecessary readmission of the patients in the hospital. Moreover, they can improve the quality of home health care services and clinical outcomes. Specific HF CPGs for home care seem more practical but can also be used in conjunction with general HF guidelines. However, what is certain is that the guidelines should be clear, concise, and practical, or even short versions can be produced from extended versions for ease of use.

As nurses are one of the largest groups of the home-based healthcare providers, it is recommended that authors of the guidelines pay more attention to the role of nurses in outpatient settings, such as patients’ home. Also, more effective education of HF patients and their families and their participation in self-care should be considered.

## Strengths and limitations

This systematic review included a comprehensive search for guidelines, the systemic and explicit application of eligibility criteria, and the careful consideration of guideline quality by using the AGREE-II, and did a rigorous analytical approach. However, several limitations could have biased our results. There is the possibility of missing clinical guidelines in other languages, as we restricted our search to only English language guidelines. AGREE-II emphasizes the technical validity of guideline recommendations, not the clinical acceptability or effectiveness. The information of this review was included particular sources at a specific range time; new guidelines have been released after May 2021, are not included.

## Conclusions

This is the first study to identify and evaluate clinical home care guidelines for HF patients. This review showed that there are 10 general and specific guidelines for home care of HF patients, but there are only two specific nursing guidelines. Two guidelines with high quality were: “NICE” and “Adapting HF guideline for nursing care in home health care settings”. It is recommended that they use by home healthcare nurses during caring of HF patients at their home.

## Future work is required to ensure:

- The incoming guidelines make suggestions on the development of viable strategies for homecare stakeholders. It is very important a person-centered approach to guideline development to ensure that all bio-psycho-social needs are addressed.
- Continuous care needs to be strengthened and effective interventions that ensure quality HF care to home care.
- A comprehensive understanding of complex needs would facilitate and evaluate the appropriateness of current health policy proposals for home care.
- It is suggested that guideline authors developed useful and holistic CPG for Home Health Care in HF Patients.
- According to the special needs and resources, cultural and economic differences in each health care system, clinical guidelines should be adapted.
- The future study has to look for impediments to guideline implementation and adherence and strategies to overcome these barriers.

## Electronic supplementary material

Below is the link to the electronic supplementary material.

Supplementary Material 1

## Data Availability

The datasets used and/or analysed during the current study available from the corresponding author on reasonable request. All requests will be answered within a maximum of 1 month by email.

## Footnotes

1 National Institute for Health and Care Excellence.

2 Ia evidence from systematic reviews or meta-analysis of randomised controlled trials. Ib evidence from at least one randomised controlled trial. IIa evidence from at least one controlled study without randomisation. IIb evidence from at least one other type of quasi-experimental study such as a cohort study. III evidence from non-experimental descriptive studies, such as comparative studies, correlation studies, case-control studies and case series. IV evidence from expert committee reports or opinions or clinical experience of respected authorities.

3 Canadian Cardiovascular Society.

4 Scottish Intercollegiate Guidelines Network.

5 1 + + High-quality meta-analyses, systematic reviews of RCTs, or RCTs with a very low risk of bias. 1 + Well-conducted meta-analyses, systematic reviews, or RCTs with a low risk of bias. 1 - Meta-analyses, systematic reviews, or RCTs with a high risk of bias. 2 + + High-quality systematic reviews of case-control or cohort studies, High-quality case-control or cohort studies with a very low risk of confounding or bias and a high probability that the relationship is causal. 2 + Well-conducted case-control or cohort studies with a low risk of confounding or bias and a moderate probability that the relationship is causal. 2 - Case-control or cohort studies with a high risk of confounding or bias and a significant risk that the relationship is not causal. 3 Non-analytic studies, e.g. case reports, case series. 4 Expert opinion.

6 European Society of Cardiology.

7 American Heart Association and American College of Cardiology Foundation.

8 United States of America.

9 Level of Evidence.

10 Level A Randomized, Controlled, Clinical Trials May be assigned based on results of a single methodologically rigorous trial. Level B Cohort and Case-Control Studies Post hoc, subgroup analysis, and meta-analysis Prospective observational studies or registries. Level C Expert Opinion Observational studies-epidemiologic findings Safety reporting from large-scale use in practice.

11 Heart Failure Society of America.

12 Institute for Clinical Systems Improvement.

## List of abbreviations

HF: Heart Failure
NYHA: New York Heart Association
