# Supplementary material for: A systematic review on clinical guidelines of home health care in heart failure patients
Source: BMC Nurs. 2023 Apr 18;22:127. doi: 10.1186/s12912-023-01294-w (PMC10111843; doi:10.1186/s12912-023-01294-w)
Supplement: Supplementary file 1 — Supplementary Material 1 [file 12912_2023_1294_MOESM1_ESM.docx]

**Search strategy**

PubMed

(Home Care Services[Mesh] OR "Home Care*" [tiab]) AND (Heart Failure [mesh] OR "Heart Failure"[tiab] OR "congestive heart failure"[tiab]) AND (guideline [mesh] OR guideline [tiab] OR "Practice Guideline"[mesh] OR Practice Guideline [tiab] OR Guideline Adherence[mesh] OR Guideline Adherence [tiab])

Web of Science

(TS= Home Care Services OR Ti= " Home Care ") AND (TS= " Heart Failure " OR TI= " Heart Failure ") AND (TS= guideline OR TI= guideline OR TS= "Practice Guideline" OR TI= "Practice Guideline" OR TS= Guideline Adherence OR TI= Guideline Adherence)

Scopus

(INDEXTERMS(Home Care Services) OR TITLE-ABS-KEY("Home Care")) AND (INDEXTERMS (Heart Failure) OR TITLE-ABS-KEY ("Heart Failure")) AND (INDEXTERMS (Guideline) OR TITLE-ABS-KEY (Guideline) OR INDEXTERMS ("Practice Guideline") OR TITLE-ABS-KEY ("Practice Guideline") OR INDEXTERMS (Guideline Adherence) OR TITLE-ABS-KEY (Guideline Adherence))

The following are search strategies for clinical practice guidelines in in Guideline Websites and Google Scholar:

(Home Care Services OR Home Care) AND (Heart Failure OR Heart Failure OR congestive heart failure) AND (guideline OR practice guideline OR Guideline Adherence)

| **Search term** | | **Synonyms in PubMed** | **Synonyms in Emtree** | **Synonyms in Paper** |
| --- | --- | --- | --- | --- |
| Component 1 | guideline  adoption | guideline [mesh]  Guideline Adherence[mesh]  Practice Guideline[mesh]  Guidelines as Topic[mesh]  Entry Terms:   - Adherence, Guideline - Policy Compliance - Compliance, Policy - Protocol Compliance - Compliance, Protocol - Institutional Adherence - Adherence, Institutional | clinical practice guidelines; guidelines; guidelines as topic; practice guidelines; practice guidelines as topic  adherence to protocol; compliance to protocol; doctor adherence; doctor compliance; doctors' adherence; doctors' compliance; guideline adherence; nurse adherence; nurse compliance; nurses' adherence; nurses' compliance; nursing adherence; nursing compliance; physician adherence; physician compliance; physicians' adherence; physicians' compliance; policy adherence; policy compliance; practitioner adherence; practitioner compliance; practitioners' adherence; practitioners' compliance; procedural adherence; procedural compliance; protocol adherence; reporting compliance; technical adherence; technical compliance | Health Planning Guidelines;;guidelines; Clinical Protocols; Consensus Development Conference |
| Component 2 | Home Care | Home Care Services [mesh]  Entry Terms:   - Home Care Service - Service, Home Care - Care Services, Home - Domiciliary Care - Care, Domiciliary - Services, Home Care - Home Care - Care, Home | domestic health care; domiciliary care; home care agencies; home care program; home care programme; home care service; home care services; home care services, hospital-based; home health care; home health nursing; home help; home nursing; home service; home treatment; homecare; homemaker services | Home Health Aides; house call |
